# Oncological and functional outcomes for transoral robotic surgery following previous radiation treatment for upper aerodigestive tract head and neck cancers. A French multicenter GETTEC group study

**DOI:** 10.1002/cam4.7031

**Published:** 2024-03-28

**Authors:** Olivier Malard, Matilde Karakachoff, Christophe Ferron, Stéphane Hans, Sébastien Vergez, Renaud Garrel, Philippe Gorphe, Lionel Ramin, Laure Santini, Alexandre Villeneuve, Audrey Lasne‐Cardon, Florent Espitalier, Audrey Hounkpatin

**Affiliations:** ^1^ Department of Otolaryngology Head and Neck Surgery Nantes University Hospital Nantes France; ^2^ Nantes Université, CHU Nantes, Pôle Hospitalo‐Universitaire 11: Santé Publique, Clinique des données, INSERM, CIC 1413 Nantes France; ^3^ Department of Otolaryngology‐Head and Neck Surgery Foch Hospital Suresnes France; ^4^ Head and Neck Surgery Department Cancer Institute Toulouse‐Oncopole Toulouse France; ^5^ Department of Head and Neck Surgery Montpellier Guy De Chauliac University Hospital Montpellier France; ^6^ Department of Head and Neck Oncology, Gustave Roussy Institute University Paris‐Saclay Villejuif France; ^7^ Department of Head and Neck Surgery Limoges Dupuytrens University Hospital Limoges France; ^8^ ENT—Head and Neck Surgery Department, La Conception University Hospital Aix Marseille University Marseille France; ^9^ Head and Neck Surgery Department, Georges‐Pompidou European Hospital Paris France; ^10^ Department of Head and Neck Surgery, François Baclesse Cancer center Normandie University Caen France

**Keywords:** head and neck cancer, radiotherapy, squamous cell carcinoma, transoral robotic surgery

## Abstract

**Background:**

Transoral robotic surgery (TORS) opens new perspectives. We evaluated the outcomes for patients having undergone TORS after previous radiotherapy.

**Methods:**

A retrospective multicenter study (*n* = 138) in a previously irradiated area between 2009 and 2020. Survival was assessed with the Kaplan–Meier method. Prognostic factors were evaluated using a chi‐squared test, Fisher's test, or Wilcoxon's test.

**Results:**

The median length of hospital stay was 12.5 days. Bleeding was the most frequent postoperative complication (15.2%, *n* = 22). Prophylactic vessel ligation did not significantly decrease bleeding. Complications were significantly lower for Tis, T1, and N0 tumors. 91.6% (*n* = 120) of the patients with a perioperative tracheotomy could be decannulated. Larynx was functional for 65.94% of the patients. The median length of follow‐up was 26 months. The 5‐year overall and relapse‐free survival rates were respectively 59.9% and 43.4%.

**Conclusion:**

Oncological and functional results confirmed the value of TORS as a treatment in previously irradiated area.

## Graphical abstract

A retrospective, multicenter study of TORS procedures (*n* = 138) for HNSCC in a previously irradiated area. The 5‐year overall and relapse‐free survival rates were respectively 59.9% and 43.4%. At last follow‐up, 65.94% of the patients had a functional larynx.

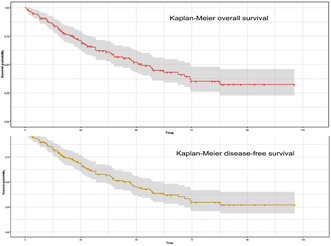

## 1 INTRODUCTION

Although surgery is the benchmark curative treatment for head and neck squamous cell carcinoma (HNSCC) in previously irradiated areas, the oncological and functional results are mediocre.^1^, ^2^, ^3^ Goodwin et al.'s meta‐analysis of 32 studies with a total of 1080 patients found a 5‐year overall survival rate after salvage surgery of 39%.^4^ Likewise, Hamoir et al. found a 5‐year overall survival rate of 42% for HNSCC (regardless of the site).^5^ Other treatments (e.g., repeat radiotherapy, with or without concomitant chemotherapy) are only possible in a few cases.^6^

Transoral robotic surgery (TORS) has now become a full part of the therapeutic arsenal for head and neck cancer.^7^, ^8^ Several studies have shown that TORS is an effective alternative to open surgery and is associated with a lower morbidity rate^9^, ^10^, ^11^, ^12^, ^13^, ^14^, ^15^; however, the application of TORS in previously irradiated areas has not been extensively evaluated.

The present retrospective multicenter study was conducted by members of the *Groupe d'Etude des Tumeurs de la Tête et du Cou* (GETTEC)'s working group on robotic surgery. We studied patients having undergone TORS for excision of an HNSCC after radiotherapy for a previous head and neck carcinoma. The study's objective was to assess the oncological and functional outcomes of TORS procedures after radiotherapy.

## 2 METHODS

### 2.1 Study design

**FIGURE 1:**
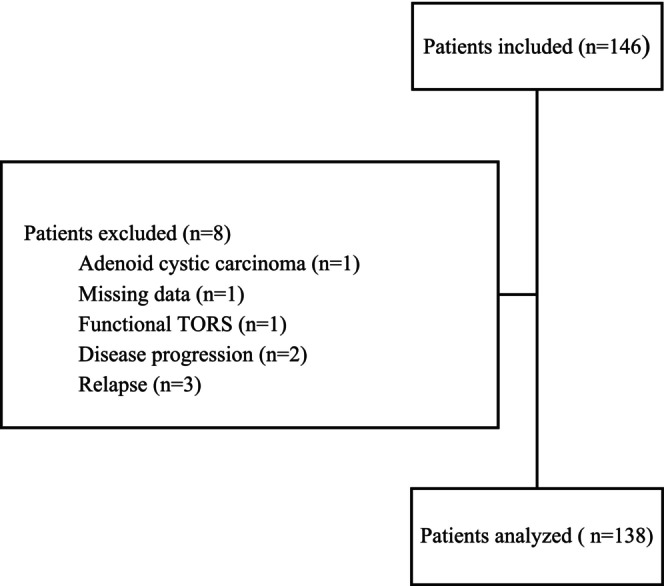
Study flow chart.

Members of the GETTEC's working group on robotic surgery conducted a retrospective, observational, multicenter study between November 2009 and March 2020. All patients having undergone TORS for HNSCC in a previously irradiated area during the study period were included, patients had to be considered as second primary metachronus tumors. Patients with other histological types, locoregional and distant progressive disease were excluded (Figure 1).

### 2.2 Surgical procedure

The indications for surgery were always discussed and approved in a multidisciplinary team meeting. Patients were provided with information on the surgery's procedures and risks.

The tumor's degree of exposure with a retractor for robotic surgery was always evaluated endoscopically prior to TORS. Surgery was performed under general anesthesia and after orotracheal or nasotracheal intubation, using different Da Vinci robot models (Intuitive Surgical Inc., Sunnyvale, CA, USA). After the retractor had been positioned, the surgeon again checked that the degree of exposure was sufficient. The lesion was excised with a 8 mm Maryland dissecting forceps and a 8 mm monopolar spatula. The procedure was observed with a 0° or 30° inclinable binocular surgical microscope.

### 2.3 Study endpoints

We collected data on the patients' characteristics prior to TORS: sex, age at the time of TORS, tobacco and alcohol consumption, p16 status, any history of pharyngolaryngeal surgery or cervical lymph node dissection, any history of locoregional radio/chemotherapy, the previous tumor sites, and the dates on which cancers were diagnosed. We also documented the tumor site targeted with TORS, concomitant lymph node dissection, procedures for mitigating volume loss, prophylactic vascular ligature, prophylactic tracheotomy, placement of a nasogastric tube, the length of hospital stay (LOS), postoperative complications, the time to oral refeeding, the time to decannulation, and the need for postoperative speech therapy.

Oncological variables were analyzed: the pTNM stage according to the Eighth Edition of the Union for International Cancer Control's Classification of Malignant Tumors (UICC TMN8), additional surgery, adjuvant radiotherapy and/or chemotherapy, tumor relapse after TORS (regional lymph nodes or metastatic cancer) and death. Excision margins according to main published data were defined as R0 if superior to 2 mm, R1 if in contact or inferior to 2 mm.^16^, ^17^, ^18^

### 2.4 Statistical analysis

A descriptive analysis of the patients' characteristics was performed using Excel® 2019 (Microsoft Corporation, Redmond, WA, USA). Quantitative variables were expressed as the mean or the median (range), and qualitative variables were expressed as the frequency (percentage).

In exploratory analyses were performed. Differences in qualitative variables were probed with a chi‐squared test or (if the sample size was below five) Fisher's exact test. Differences in continuous variables were probed with a *t*‐test or (if the data were not distributed normally) Wilcoxon's test. Prognostic factors for postoperative complications were considered prior to pharyngolaryngeal surgery or cervical lymph node dissection, time interval between radiotherapy and TORS, first‐line chemoradiotherapy, tumor site, and lymph node dissection during the TORS session. The groups' overall and relapse‐free survival rates were estimated using the Kaplan–Meier method. Data were compared in a Cox proportional hazards modeling for multivariable analysis statistical model (time‐to‐event) outcome as the probability that the event of interest (death) occurred before *t*. All tests were two‐tailed, and the threshold for statistical significance was set to *p* < 0.05. R software (version 3.6.3) was used for statistical analyses.

## 3 RESULTS

### 3.1 Patients and procedures

This study is one of the largest multicentric evaluation of TORS treatment for patients after irradiation for a previous primary head and neck cancer. Missing data regarding to HPV status, reconstructive technique, feeding tube are issues responsible as shortcomings in the manuscript, as number of patient in different tumor site localization.

The median duration of follow‐up was 26 months (0–127). The median (range) age was 62 (44–86), 85.51% of the patients were men, and 93.48% of the patients were former or current smokers. At the time of the TORS, 76.81% of the study participants were still smoking. With regard to alcohol, 71.74% had a history of abuse and 62.32% were still alcohol dependent.

Sixty‐six patients (47.83%) had already undergone surgery of the oral or pharyngolaryngeal cavity, and 62 (44.93%) had already undergone cervical lymph node dissection.

At the time when TORS was indicated, six (4.35%) patients had a gastrostomy tube and eight (5.80%) had already been tracheotomized or laryngectomized. One of the patients with a gastrostomy tube had also been laryngectomized (Table 1).

**TABLE 1:** Characteristics of the study participants (*N* = 138).

	*n* (%) or mean [median] (range)	
Patient characteristics		
Men	118 (85.51%)	
Women	20 (14.49%)	
Age (years)	62.08 [62] (44–86)	
Risk factors		
Ongoing tobacco use	129 (93.48%)	
Former smokers	23 (16.67%)	
Ongoing alcohol abuse	99 (71.74%)	
Previous alcohol abuse	13 (9.42%)	
Positive p16 status	14 (10.14%)/NA: 79 (57.25%)	
Treatment with an antiaggregant or anticoagulant	43 (31.16%)	
	NA: 30 (21.74%)	
Previous cancer history		
History of pharyngolaryngeal/oral cancer surgery	67 (48.55%)	
History of cervical lymph node dissection	62 (44.93%)	
History of concomitant radiochemotherapy	83 (60.15%)	
Mean number of previously treated tumor sites per patient	1.31	
Tumor sites previously treated with radiotherapy	150	
	Oral cavity: 25 (16.67%)	
	Oropharynx: 61 (40.67%)	
	Hypopharynx: 26 (17.33%)	
	Larynx: 30 (20.00%)	
	Nasopharynx: 1 (0.67%)	
	Esophagus: 5 (3.33%)	
	Tx tumor: 2 (1.33%)	
TNM stages of the sites previously treated with radiotherapy	*N* = 150	
	T1N0: 2 (1.33%)	T3N0: 8 (5.33%)
	T1N1: 1 (0.67%)	T3N1: 4 (2.67%)
	T1N2: 4 (2.67%)	T3N2: 1 (0.67%)
	T2N0: 7 (4.66%)	T4N0: 1 (0.67%)
	T2N1: 4 (2.67%)	T4N2: 2 (1.33%)
	T2N2: 4 (2.67%)	TxN1: 2 (1.33%)
	T2N3: 2 (1.33%)	NA: 108 (72.00%)
Repeat radiotherapy before TORS	6 (4.35%)	

Abbreviation: NA, not available.

According to UICC TMN8, 122 cancers (88.41%) were stage T1 or T2, and 29 (21.01%) were stage III or IV: these classifications were mainly prompted by lymph node invasion (Table 2).

**TABLE 2:** The CTNM stage, according to UICC TMN8.

	‐	N0	N1	N2	Total	%
Tis	4				4	2.9
T1		57	2	1	60	43.48
T2		48	10	4	62	44.93
T3		2	4	0	6	4.35
T4a		3	3	0	6	4.35
Total	4	110	19	5	138	100
%	2.90	79.71	13.77	3.62	100	

Stage	*N*	%
Stage 0	4	2.9
Stage I	57	41.31
Stage II	48	34.78
Stage III	18	13.04
Stage IV	11	7.97

The anatomic distribution for TORS was as follows: oral cavity, *n* = 2 (1.4%); oropharyngeal, *n* = 84 (60.4%); hypopharyngeal, *n* = 25 (18%), and laryngeal, *n* = 28 (20.1%). In one patient, two distinct lesion sites were treated during the same procedure. There were two conversions to an external approach because a lack of exposure prevented excision of the whole tumor.

The cervical lymph nodes were removed during the TORS in 49 of the 139 patients (35.3%) and after the TORS in two patients. Prophylactic intraoperative tracheotomy was performed in 57 cases (41.3%). Reconstruction was performed in 39 cases (28.3%), with a free flap used in 35 (an anterolateral thigh flap: *n* = 17; an antebrachial flap: *n* = 14; a medial sural perforator flap: *n* = 2; type not specified: *n* = 2) and a pedicle flap in the other four (a facial artery musculomucosal flap: *n* = 1; nonspecified flap: *n* = 3).

Additional or adjuvant treatment was administered in 17 cases (12.3%). Thirteen patients (9.4%) underwent re‐irradiation, immunotherapy or chemotherapy. The indications for re‐irradiation were one or more histolopathologic criteria for a poor prognosis: narrow margins or margin invasion in 11 cases (8%), vascular emboli and/or perineural invasion in four (2.9%), and more than one lymph node invaded and/or capsular invasion in three (2.2%). Repeat radiotherapy alone was performed in four cases (2.9%). Further surgery was performed in three cases (2.2%), including one total laryngectomy as salvage treatment for cancer (Table 3).

**TABLE 3:** The surgical procedures (*N* = 138).

	*n* (%) or mean [median] (range)
Site tumors	*n* = 139
	Oral cavity: 2 (1.44%)
	Oropharynx: 84 (60.43%)
	Hypopharynx: 25 (17.99%)
	Larynx: 28 (20.14%)
Cervical lymph node dissection	51 (36.96%)
Tracheotomy	57 (41.30%)
Prior tracheotomy or laryngectomy	8 (5.80%)
Conversion to an external approach (due to insufficient exposure)	2 (1.45%)
Reconstruction	39 (28.26%)
Prophylactic intraoperative vessel ligation	26 (18.84%)
	NA: 12
Intraoperative nasogastric tube placement or gastrostomy	87 (63.04%)/3 (2.17%)
	NA: 11
Prior gastrostomy	6 (4.35%)
Additional or adjuvant treatment	17 (12.32%)
Further surgery (total laryngectomy)	1 (0.72%)
Further surgery and radiotherapy	1 (0.72%)
Further surgery and chemotherapy	1 (0.72%)
Repeat radiotherapy only	4 (2.90%)
Concomitant chemoradiotherapy	5 (3.62%)
Concomitant chemoradiotherapy and immunotherapy	1 (0.72%)
Radiotherapy and immunotherapy	1 (0.72%)
Chemotherapy	2 (1.45%)
Immunotherapy	1 (0.72%)

Abbreviation: NA, not available.

### 3.2 Functional outcomes and complications

The median (range) LOS was 12.5 days (0–428) (Table 4). Two patients died of postoperative complications. The first died on postoperative day (POD) 41, due to a lung disorder complicated by septic shock and then multi‐organ failure. The second died 3 months after surgery, due to an epidural infection that spread from a focus of spondylodiscitis at the surgical site.

**TABLE 4:** Postoperative data (*N* = 138).

	*n* (%) or mean [median] (range)
Length of hospital stay (days)	21.84 [12.5] (0–428)
	*n* = 131
Intraoperative nasogastric tube/gastrostomy	87 (66.41%)/3 (2.29%) unknown: 11
Gastrostomy during follow‐up	
Time to gastrostomy (months)	53 (40.46%)
Oral refeeding	6.98 [1] (0–67)
Time to oral refeeding (days)	120 (91.60%)
	29.87 [8.0] (0–436)
Aspiration	55 (39.86%)
Lung disease or disorder	24 (17.39%)
Rehabilitation for swallowing/speech therapy	92 (66.67%)
	*N* = 130
Intraoperative prophylactic tracheotomy	57 (43.85%)
Therapeutic tracheotomy within 3 months of surgery	4 (3.08%)
Decannulation/number of tracheotomies	54/59 (91.5%)
Time to decannulation (days)	24.63
Occurrence of at least one surgery‐related complication Death	36 (26.09%) 2 (1.45%)
Bleeding	21 (15.22%)
Dyspnea	4 (2.90%)
Cervical hematoma	11 (7.97%)
Pharyngostoma	2 (1.45%)

Abbreviation: NA, not available.

Twenty‐one patients (15.22%) had postoperative bleeding; of these, 13 resolved without treatment, five required further surgery, two underwent interventional radiology (arterial embolization), and one underwent packing and tracheotomy. No bleeding‐related neurological complications or deaths were noted. Two of these 21 patients (9.52%) had undergone intraoperative prophylactic vessel ligation. The absence of prophylactic vessel ligation was not significantly associated with the likelihood of bleeding (*p* = 0.364) in this study, with the limitation of the number of patients.

Furthermore, 11 of these 21 patients had a prophylactic tracheostomy tube placed during the TORS procedure. Only one patient was tracheotomized because of bleeding.

Of the 51 (36.96%) patients having undergone cervical lymph node dissection, 10 presented a cervical hematoma. There was one instance of cervical hematoma in a patients not having undergone cervical lymph node dissection.

Two pharyngolaryngeal fistulae were recorded; one of the patients had a laryngeal T2N0 cancer and had not undergone cervical lymph node dissection, whereas the other had an oropharyngeal T2N0 cancer and had undergone concomitant ipsilateral cervical lymph node dissection. Neither of these two patients had flap coverage.

In the subgroup of patients not having undergone tracheotomy or tracheostomy before TORS, secondary tracheotomy was required in four cases (3.08%), two of whom had been decannulated after their intraoperative tracheotomy. The indications were postoperative dyspnea in three of the cases and bleeding in the last. Of the 59 patients tracheotomized during or after TORS, 54 were decannulated after a median (range) of 13.5 days (3–375). The other five patients died within 16 months of surgery and had not been decannulated.

Fifty‐five (39.9%) patients experienced episodes of laryngeal aspiration, 24 (17.4%) developed a lung disorder, and 92 (66.7%) required speech therapy after discharge. Data from the six patients fed enterally prior to TORS and from the patient, who died on POD 41 were excluded from the analysis of oral refeeding. At least partial oral refeeding was possible for 120 (91.6%) of the 131 subjects with data, after a median of 8 days. Fifty‐three patients (40.5%) required a gastrostomy during the follow‐up period, after a mean time interval of 6.98 months.

In the exploratory analyses, patients graded as Tis, T1 and N0 had a significantly lower postoperative complication rate (death, dyspnea, bleeding, hematoma, and infection). In contrast, age, previous treatments, and the tumor site were not significantly associated with the risks of death or bleeding. Age ≥ 64, a laryngeal tumor, and previous ENT surgery were significantly associated with episodes of aspiration, and age ≥ 66 and T2 status were significantly associated with the development of a postoperative lung disorder.

### 3.3 Oncologic outcomes

In the definitive histopathologic assessment, 110 patients were graded as R0 (79.71%). Intraoperative margin assessment was performed in 102 cases (73.91%).

**FIGURE 2:**
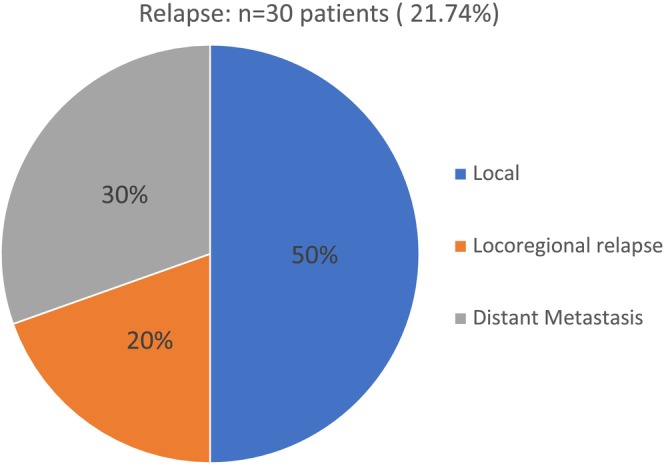
The site of relapse.

During a median (range) follow‐up period of 26 months (0–127), 30 (21.74%) patients relapsed (Figure 2). The median time to relapse was 10 months. Eight patients developed HNSCC at a second site (5.80%). Fourteen patients (10.15%) developed another type of cancer at another site (bronchial or pulmonary site in seven of cases).

**FIGURE 3:**
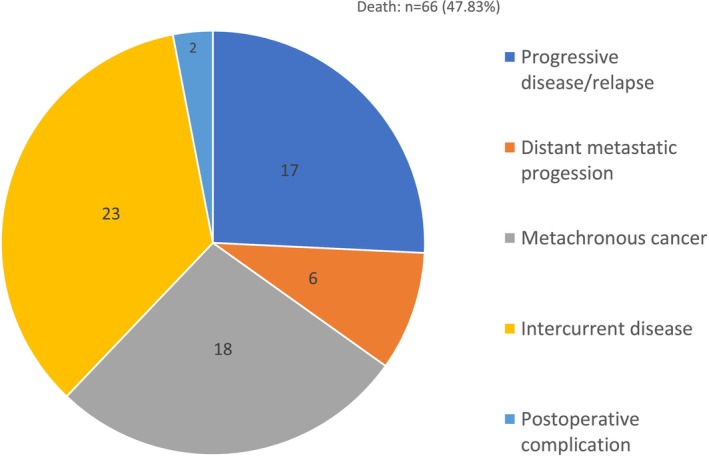
Causes of death during the follow‐up period.

Sixty‐six (47.83%) patients died during the follow‐up. Two of the deaths resulted from postoperative complications (Figure 3).

**FIGURE 4:**
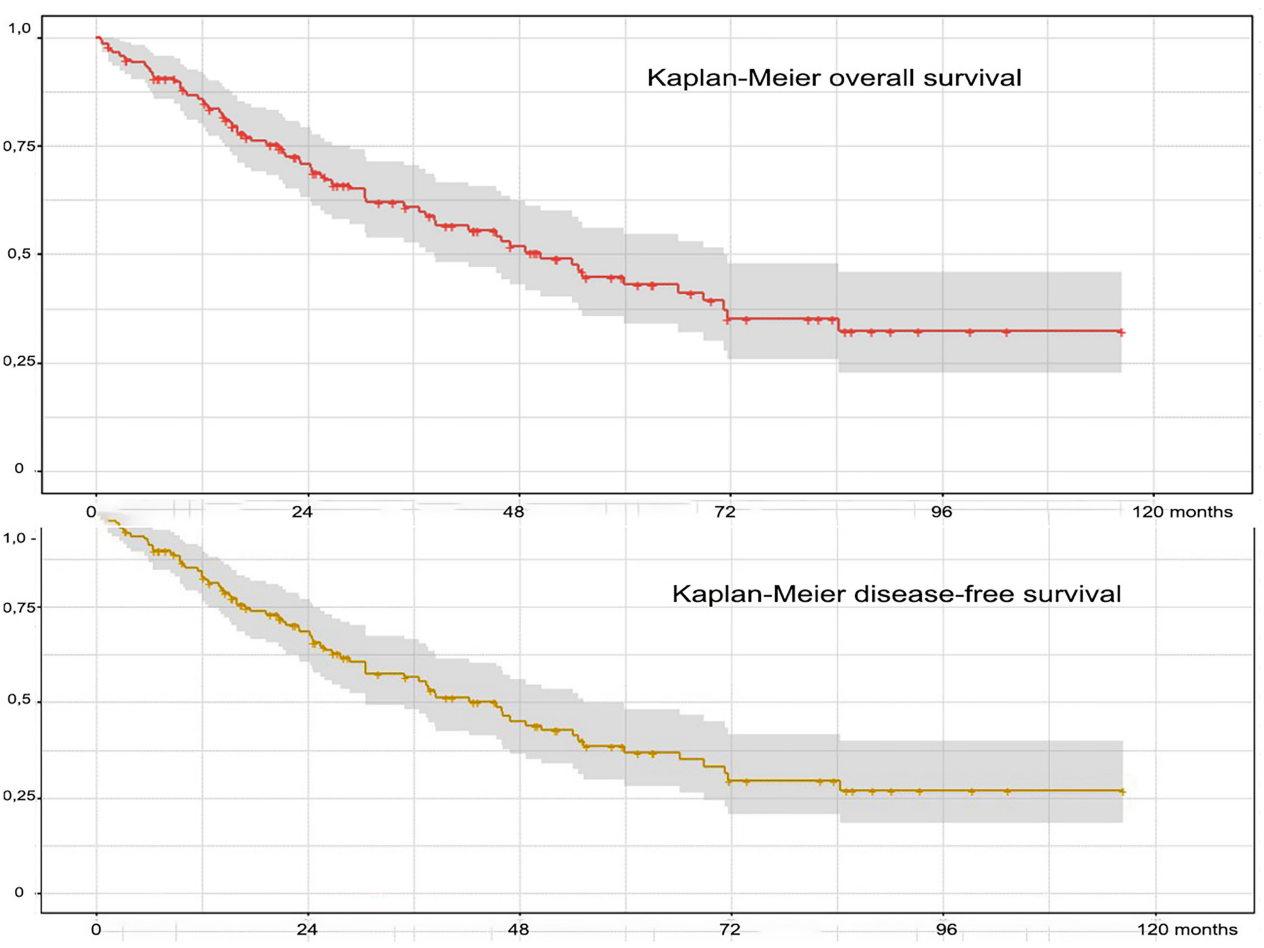
Kaplan–Meier plots for overall survival and disease‐free survival.

According to the Kaplan–Meier analysis, the overall and relapse‐free survival rates were respectively, 72.20% and 69.10% at 2 years and 59.90% and 43.40% at 5 years (Figure 4).

In a univariate Cox model, older age, higher TNM stage, R1 surgical margins, and previous ENT surgery for cancer were significantly associated with a higher likelihood of death. Conversely, cervical lymph node dissection during the TORS procedure and chemotherapy at the same time as the initial radiotherapy were associated with a significantly higher overall survival rate. The cohort dating back to 2009 limited statistical HPV data availability.

A multivariate analysis confirmed that the TNM stage and lymph node dissection were associated with overall survival. A time interval of more than 3 years between radiotherapy and TORS was associated with longer overall survival. The tumor site and any additional treatments after TORS were not significantly associated with overall or relapse‐free survival.

At last follow‐up, 91 (65.94%) patients had a functional larynx (i.e., no tracheotomy or tracheostomy) and had not needed gastrostomy. Oral feeding was noted for 96 patients (69.57%). In the exploratory analyses, age ≥ 64 and the time interval of more than 4 years between radiotherapy and TORS were significantly associated with long‐term laryngeal function.

## 4 DISCUSSION

The present study of oncological and functional outcomes evaluated the place of TORS in the treatment of HNSCC after radiotherapy in patients with adequate transoral access and lower volume tumors. It was conducted by members of the GETTEC's working group on robotic surgery, which ensured that surgical procedures did not vary greatly form one center to another. Most centers followed the French ENT society's 2017 guidelines.^19^

The postoperative morbidity rate for TORS in irradiated area was moderate. Our postoperative complication rate was 26.1%. Hay et al.^20^ reported a rate of 35% among 122 patients. Hardman et al. in the RECUT study (*n* = 278) evaluated the outcomes of TORS with median follow‐up of 38.5 months and found 11% of complications. Turner et al. found in a systematic review that the rate of death following salvage TORS was 3.6%, whereas Hardman et al. a 30‐day mortality of 1.8%.^21^

The complication rate was significantly lower for Tis, T1, and N0 forms. The extent of the resection (directly correlated with the T stage) therefore appears to be the main predictor of complications. Hay et al. also highlighted this factor. Significant losses of substance can be complicated by blood vessel exposure and bleeding, delayed wound healing, and fistulae requiring coverage with a local or free flap.^22^

The most frequent described complication is bleeding,^20^, ^23^, ^24^ between 6 to 18.5%. For Hardman et al. in the RECUT study, hemorrhages with return to theater was seen in 8.1%.^21^ In the systematic review of Turner et al., major hemorrhage rate (requiring operative intervention) was 10.5% and minor hemorrhage rate (treated with observation and/or transfusion) was 6.3%.^25^ In our study in irradiated areas, the bleeding rate of 15.22% was within the range reported in the literature. The absence of prophylactic vascular ligature during TORS was not associated with an excess risk of bleeding in our data. According to Gleysteen et al.^23^ and Kubik et al.,^26^ artery ligation had no influence on the overall incidence of bleeding but reduced the severity of hemorrhage, whereas repeat radiotherapy prior to TORS was a risk factor for severe bleeding. Reconstruction was associated with lower overall hemorrhage rate, but had no impact on major postoperative hemorrhage rates.^25^ Thus, use of reconstruction flap and vessel ligation should be considered depending on the extent of resection and area of prior radiation when prophylactic salvage TORS is considered even if no obvious recommendation exist.

The incidence of intraoperative prophylactic tracheotomy was 43.85%. Furthermore, four patients underwent secondary tracheotomy because of a postoperative complication (dyspnea or bleeding). The overall decannulation rate was 91.5%, which is close to the value of 92.1% reported by Park et al. (*n* = 38 patients) treated with TORS for non‐irradiated hypopharyngeal cancers (all of whom had undergone prophylactic tracheotomy).^27^ According to RECUT study at 1 year, 10.8% of patients always required tracheostomies.^21^

White et al. performed a case–control study comparing transoral and external approaches for 122 patients with a relapsed oropharyngeal tumor, 88% of whom had a history of radiotherapy.^28^ TORS was associated with a significantly lower mean LOS (3.8 days, vs. eight for external approaches) and a shorter time to oral refeeding. These functional outcomes appear to be even more encouraging than in our study, where the mean LOS was around 22 days. In a comparative analysis of 34 supraglottal laryngectomies performed with TORS (*n* = 17) or cervicectomy (*n* = 17), Park et al. reported that in the absence of an external approach, the extrinsic laryngeal musculature is spared, this translated into better functional recovery, with a significantly lower LOS, a lower tracheotomy rate, and earlier resumption of oral feeding.^29^ The robot offers the operator more degrees of freedom, provides an accurate three‐dimensional view of the lesion, and thus makes it possible to remove the tumor, while sparing the healthy mucosal tissue.^29^

With previously irradiated tissue, the endoscopic approach is likely to limit the occurrence of specific complications like osteoradionecrosis, pseudarthrosis, delayed wound healing, and poor esthetic outcome.^30^ As a result, TORS improves the patient's quality of life.^31^ The emergence of TORS has therefore limited the indications for “obliterative” surgical techniques, such as oropharyngectomy with mandibulotomy.^32^ Nevertheless, two conversions to an external approach (due to insufficient exposure) were noted in our study. The use of a retractor designed for robotic surgery during the initial endoscopy avoids (albeit not fully) exposure problems.

Several researchers have addressed the question of cancer‐free margins in TORS because this is a major prognosis factor for local control.^33^ Thanks to its endoscopic nature, TORS might generate narrower excision margins than “open” surgery does. Resection with a monopolar spatula can burn the margins and lead to tissue retraction (which further accentuates the damage caused by the previous radiotherapy) and then further retraction due to formol fixation. Furthermore, the definition of margins in TORS is subject to debate and might require the anatomic location to be taken into account.^16^, ^18^, ^34^ In Hinni et al.'s series of 128 patients having undergone transoral excision of a tonsillar cancer,^35^ a mean margin width of 1.98 mm led to a 5‐year local control rate of 99%. In the RECUT study, narrow surgical margins (<1.0 mm) were statistically significantly associated with lower overall survival.^21^ In the present study, 20.29% of the margins were positive, i.e., ≤ 2 mm. This value falls within the range (0%–33%) found in the literature on large series of TORS procedures (essentially for first‐line treatment of HNSCC), as reviewed by Hamzany et al.^36^ These margin rates were associated with high levels of local control (91%–100%). Our local control rate in an irradiated area was lower, with relapse observed in 21.7% of the patients.

White et al. compared TORS with standard open surgical for recurrent cancers of the oropharynx only^28^: 88% of the surgeries concerned patients in irradiated area. This study demonstrated that TORS offers an alternative surgical approach to recurrent tumors of the oropharynx with acceptable oncologic outcomes and better functional outcomes than traditional open surgical approaches. De Almeida et al.'s cohort study included 410 TORS procedures^37^ at all HNSCC sites (as in the present study): the 2‐year overall survival rate (91%) was higher than ours but only 11 (2.7%) of the patients had already undergone radiotherapy. In the retrospective analysis of Hardman et al, the 2‐year and 5‐year outcomes were 69.0% and 62.2% for local control, 71.8% and 49.8% for overall survival.^21^

Even if our patient population was large (*n* = 138) and appeared to be representative (in terms of age and the tumor site distribution) many limits appears that constraint the strength of this results: length of follow‐up varied and availability of P16 status was limited. Flaps reconstruction for equivalent T stage was inconstant and vessels ligation were not homogenized in patients. Delay following previous radiotherapy was variable. Although the limits and the shortcomings of this retrospective study, this body of data suggests that salvage TORS procedures after radiotherapy are associated with a degree of oncological control that is at least as good as that obtained with salvage surgery with an external approach, while delivering better short‐ and long‐term functional outcomes.

## 5 CONCLUSION

Our retrospective multicenter analysis of data from 138 patients having undergone TORS for a second HNSCC in a previously irradiated area showed that the oncological and functional outcomes in this indication were acceptable. The results of this multicenter study confirmed the role of TORS for the excision of HNSCC after radiotherapy. The prognostic value of several parameters or procedures (operative vascular ligature, R0 margin accuracy, and use of a flap for coverage/reconstruction) remains to be defined.

## AUTHOR CONTRIBUTIONS

**Olivier Malard:** Conceptualization (lead); writing – original draft (lead); writing – review and editing (lead). **Matilde Karakachoff:** Methodology (supporting). **Christophe Ferron:** Data curation (equal). **Stéphane Hans:** Data curation (equal). **Sébastien Vergez:** Data curation (equal). **Renaud Garrel:** Data curation (equal). **Philippe Gorphe:** Data curation (equal). **Lionel Ramin:** Data curation (equal). **Laure Santini:** Data curation (equal). **Alexandre Villeneuve:** Data curation (equal). **Audrey lasne‐cardon:** Data curation (equal). **Florent Espitalier:** Data curation (equal). **Audrey Hounkpatin:** Formal analysis (equal); methodology (equal); writing – original draft (equal).

## FUNDING INFORMATION

This research did not receive any specific funding from agencies or organizations in the public, commercial, or not‐for‐profit sectors.

## ETHICS STATEMENT

The study has been conducted in full accordance with ethical principles, including the World Medical Association Declaration of Helsinki (version 2002) and in accordance with French legislative and regulatory requirements, and the European Good Clinical Practice directive (2005/28/EC) by Groupe nantais étique dans le domaine de la santé (GNEDS). Written informed consent was obtained.

## Supporting information


Data S1:

## Data Availability

The data that support the findings of this study are available in the supplementary material of this article.
